# Local radiotherapy alone following neoadjuvant chemotherapy and surgery in combined clinical stage II and III breast cancer

**DOI:** 10.1186/s13014-016-0670-2

**Published:** 2016-07-26

**Authors:** Rohen White, Tamara Dinneen, Andreas Makris

**Affiliations:** 1 University of Western Australia, Nedlands, Australia; 2 Breast Cancer Research Unit, Mt Vernon Cancer Centre, Middlesex, UK

**Keywords:** Breast cancer, Neoadjuvant chemotherapy, Regional radiotherapy, Patterns of recurrence

## Abstract

**Purpose:**

The outcomes and recurrence patterns for patients with combined clinical stage II and III breast cancer treated with local but not regional radiotherapy after neoadjuvant chemotherapy (NAC) and surgery are poorly documented.

**Methods:**

We performed a retrospective review of a prospectively collected database comprised of breast cancer patients who received NAC at our institution. 172 patients met the specified criteria of receiving NAC, surgery inclusive of axillary nodal dissection and post-operative local (but not regional) radiotherapy.

**Results:**

One hundred eleven patients (64.5 %) were of combined clinical stage II and 61 (35.5 %) stage III at diagnosis. 103 patients (59.9 %) were clinically node positive with 101 cN1. On post-NAC pathology 29 (16.9 %) patients had a complete response, 30 (17.6 %) were combined yp stage I, 104 (60.5 %) yp stage II and 9 (5.2 %) yp stage III. 77 (44.8 %) were node positive on post-NAC pathology, all ypN1. 52.3 % were treated with breast conservation. At a median follow up of 67 months, 56 patients experienced breast cancer recurrence and 47 had died with breast cancer the dominant cause. Actuarial 5 and 10 year estimated freedom from locoregional recurrence (FFLRR), freedom from distant metastases (FFDM), disease free (DFS) and overall survival (OS) were 90 and 83.5, 74.5 and 64, 69.5 and 56, 79.5 and 65 % respectively. The most common pattern of failure was distant alone (without local or regional failure). Regional failure as the only site of first failure occurred in just three patients but was a component of first failure in a further twelve. Predictive factors on multivariate analysis for FFLRR were clinical stage II and estrogen receptor positivity. Prognostic factors were ypN0 stage and estrogen receptor positive status.

**Conclusions:**

Local radiotherapy alone may be reasonable for selected patients. Isolated distant recurrence is the dominant mode of failure for breast cancer patients who have received local radiotherapy without regional coverage following NAC.

## Background

Clinical indications for radiotherapy and target volumes following neoadjuvant chemotherapy (NAC) in the treatment of breast cancer are unclear [1–3]. Randomised controlled trial results from a non-NAC setting are often extrapolated to form the basis of radiotherapy recommendations but there is accumulating non-randomised evidence that this may result in over-treatment and unnecessary toxicity [4–6]. The uncertainty regarding the place of post-operative radiotherapy is highlighted in a patterns of management report from a recently published randomised controlled trial demonstrating much variation regardless of clinical or post NAC pathological stage [7].

At our centre practice is also not uniform. Post-operative radiotherapy following NAC and surgery for breast cancer is made on an individualized basis with many clinical oncologists adopting a local radiotherapy only approach to the conserved breast or chest wall. The rationale being that the risk of residual, microscopic regional disease post NAC in those with limited or no nodal disease on pathology is sufficiently low that it may be outweighed by the potential morbidity of regional radiotherapy.

The purpose of this study was to describe actuarial rates of recurrence from a breast cancer patient population treated with NAC, radical surgery and local radiotherapy to the conserved breast or chest wall. Recurrence patterns are detailed as well as predictive factors for freedom from locoregional recurrence (FFLRR) and overall survival (OS).

## Methods

We conducted a retrospective analysis of a prospectively collected, single institution, NAC breast cancer database. Recruitment and data collection occurred between January of 1994 and December of 2013. All patients were retrospectively staged using the American Joint Committee on Cancer (AJCC) Staging Manual v7 [8]. For the purpose of the study the database was restricted to females of combined clinical stage II or III who received a breast conservation surgery or mastectomy inclusive of axillary node dissection, and received either chest wall or whole breast radiotherapy without dedicated regional radiotherapy. Patients were excluded if a breast cancer recurrence occurred prior to completion of adjuvant radiotherapy.

Within the database there is heterogeneity regarding systemic therapy regimens and much patient data pre-dated routine human epidermal receptor 2 (HER2) amplification testing. In general, staging investigations, surgery and radiotherapy were consistent across the time period of patient recruitment.

Potential axillary involvement was clarified with ultrasound guided fine needle aspirate prior to NAC with minimal use of pre-NAC sentinel node biopsy. A level I and II axillary node clearance was standard after NAC.

Local breast or chest wall radiotherapy technique and dose prescriptions over the data collection period largely conformed to that subsequently described in the United Kingdom Standardisation of Breast Radiotherapy randomised controlled trial B which commenced accrual in 1999 [9]. Patients were simulated in the supine position (chest wall) or with a slight incline (whole breast) and the radiotherapy field edges marked 1.5-2 cm from the edge of breast tissue. In the setting of mastectomy, the contralateral breast was used to estimate breast landmarks and field edges. Earlier cases used 2D techniques without simulation computed tomography (CT) but overtime CT became mandatory. There was no use of contoured target volumes during the study period. Medial and lateral tangential, parallel opposed megavoltage beams of 6 or 10 megavoltage energy were used with dose prescribed to a point halfway between the lung and the skin surface on the perpendicular bisector of the posterior beam edge. Wedges were utilised to improve dose homogeneity. In CT plans unnecessary heart and lung were shielded with multi-leaf collimators and dose homogeneity optimised with the use of field-in-field techniques. Tangent arrangements were considered acceptable if there was less than 3 cm maximal lung and 1 cm maximal heart depth in-field.

Tumour bed boost prescription was clinician dependant and delineated at the time of simulation based on palpation of the surgical defect and position of the scar. Electrons were prescribed to D-max and a suitable energy chosen such that the 90 % isodose was predicted to cover the tumour bed. The standard dose-fractionation schedules utilised in the United Kingdom over the database collection period were 40 Gy in 15 fractions and 50 Gy in 25 fractions to the chest wall or whole breast, treating once daily, five times per week. Tumour bed boost doses ranged from 10 Gy in 4 fractions to 16 Gy in 8 fractions and were also delivered once daily on consecutive weekdays. The use of chest wall bolus post mastectomy was clinician dependent and hence variable. Patients were restaged on suspicion of recurrence and all regions of recurrent disease documented as components of first recurrence.

Locoregional recurrence (LRR) was defined as the appearance of disease at one or more ipsilateral axillary, internal mammary or supraclavicular fossa nodal stations and/or ipsilateral chest wall or intact breast. This was further split into local (LR) and regional recurrence (RR). Distant metastasis (DM) was the appearance of breast cancer at any site outside of that considered LRR. Time-to-event endpoints were measured in months from diagnosis. Freedom from locoregional recurrence (FFLRR), freedom from distant metastasis (FFDM), disease free survival (DFS) and overall survival (OS) were estimated using reverse Kaplan Meier methods. Cox regression analysis was performed to assess for predictive and prognostic factors using a *p* value below 0.05 as representing statistical significance. All statistical tests were performed on SPSS version 22.0.0.0 (IBM Corporation, Armonk, New York 2013).

## Results

### Patient, tumour and treatment characteristics

The database contained 713 patients of which 172 met the criteria for inclusion. The majority of exclusions were for clinical stage I disease or the utilisation of regional radiotherapy. Table 1 describes the patient, tumour and treatment characteristics. The median patient age at diagnosis was 49 years (range 27, 86).

**Table 1:** Patient, tumour and treatment characteristics

Characteristic		Number (total = 172)	%
Age	Median (range)	49 (27, 86)	
Age group	<50	92	53.5 %
	> = 50	80	46.5 %
Laterality	Right	92	53.5 %
	Left	80	46.5 %
Breast location	Outter quadrant	95	55.2 %
	Inner and/or central	59	34.3 %
	Unknown	18	10.5 %
Clinical TNM stage^a^			
cT stage	0-2	52	30.2 %
	3	91	52.9 %
	4	29	16.9 %
cN stage	0	69	40.1 %
	1	101	58.7 %
	2	2	1.2 %
combined c stage	II	111	64.5 %
	III	61	35.5 %
Inflammatory	No	153	89.0 %
	Yes	19	11.0 %
Histology	Ductal carcinoma	138	80.2 %
	Lobular carcinoma	18	10.5 %
	Other	16	9.3 %
Grade	1	8	4.7 %
	2	91	52.9 %
	3	73	42.4 %
ER status	Negative	64	37.2 %
	Positive	101	58.7 %
	Unknown	7	4.1 %
HER2 status	Negative	81	47.1 %
	Amplified	33	19.2 %
	Unknown	58	33.7 %
NAC regimen	Anthracycline	97	56.4 %
	Taxane	16	9.3 %
	Both	54	31.4 %
	Neither	5	2.9 %
Surgery type	Lumpectomy	90	52.3 %
	Mastectomy	82	47.7 %
Axillary nodes	median (range)	12 (1, 28)	
	<9 nodes	39	16.7 %
Pathological TNM stage^a^			
yp T stage	0-2	154	89.5 %
	3	13	7.6 %
	4	2	1.2 %
	unknown	3	1.7 %
yp N stage	0	95	55.2 %
	1	77	44.8 %
combined yp stage	pCR	29	16.9 %
	I	30	17.4 %
	II	104	60.5 %
	III	9	5.2 %
>1 node positive	No	138	80.2 %
	Yes	34	19.8 %
Trastuzumab use	No	146	84.9 %
	Yes	26	15.1 %
Radiotherapy schedule	40/15	158	91.9 %
(Gray/# fractions)	50/25	11	6.4 %
	other	3	1.7 %

^a^TNM stage as per AJCC staging manual v7

Most patients received an anthracycline based NAC regimen without a taxane (56 %), nine percent received a taxane based regimen without an anthracycline and 31 % percent received both. Two thirds of patients had HER-2 testing. Approximately 51 % of patients were treated with breast conservation surgery inclusive of axillary dissection, 2 % axillary dissection alone (for a putative primary) and the remainder modified radical mastectomy. The median number of nodes removed at axillary dissection was 12 (range 1, 28).

On pre-NAC clinical staging all patients were either combined stage II or III. Only 2 patients (1.2 %) had clinical nodal stage greater than one. 16.9 % of patients achieved a complete pathological response (pCR) having no microscopically identifiable invasive tumour in breast or axillary nodal tissue. The presence of ductal carcinoma in-situ only was considered a pCR. Whilst 35.5 % were combined clinical stage III pre-NAC, only 5.7 % were combined pathological stage III suggesting widespread downstaging. All patients were yp nodal stage 0 (55 %) or 1 (45 %).

The median follow-up for the group was 67 months (range 2, 248).

### Patterns of recurrence

**Fig. 1:**
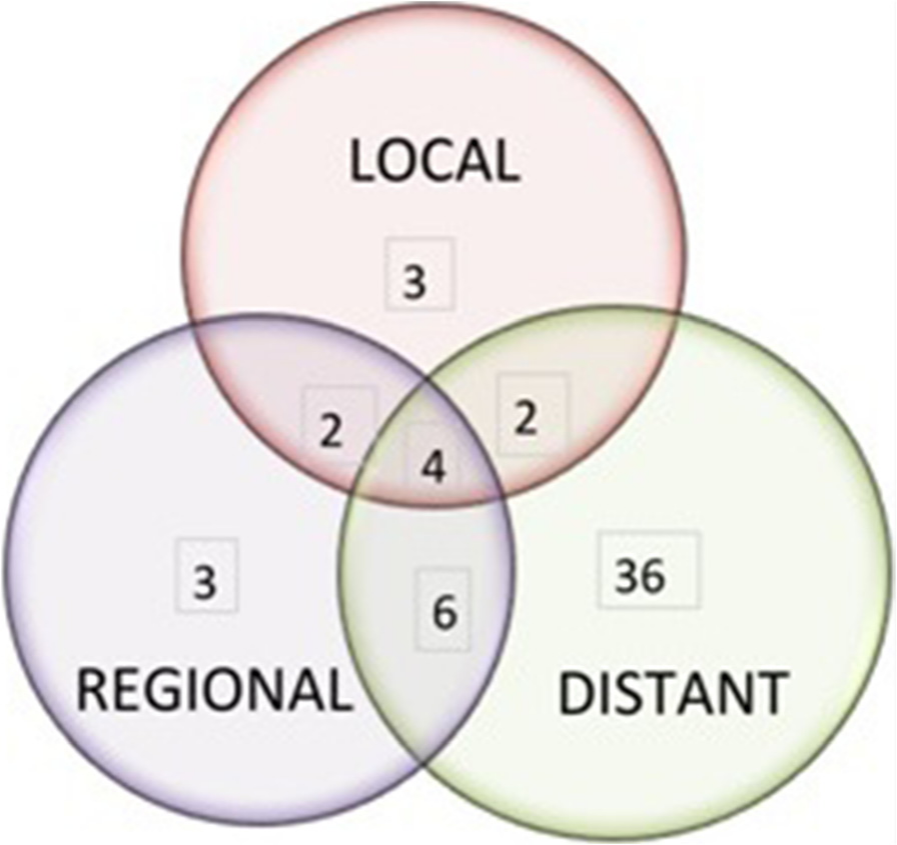
Patterns of recurrence. 56 patients experienced recurrence. Local, regional and distant recurrence were a component of first failure in 11 (6.4 %), 15 (8.7 %) and 48 (27.9 %) patients respectively

Fifty-six patients experienced recurrence at median time 30 months (range 47 to 153). The patterns of recurrence are presented in Fig. 1. LR, RR and DM were a component of first failure in 11 (6.4 %), 15 (8.7 %) and 48 (27.9 %) patients respectively. DM as the only site of failure was the most dominant pattern of failure, responsible for 36 events.

Isolated LRR occurred in eight patients of which isolated RR occurred in three, isolated LR in two and combined LR with RR in a further three. RR as a component of first failure occurred in a further 12 patients. Multiple regional sites of first failure occurred in five patients and overall the supraclavicular fossa (SCF), axilla and internal mammary nodal basins were components of first failure in 9, 6 and 5 patients respectively. Of the three isolated regional failures all involved the SCF with one additionally involving the axilla.

### Freedom from locoregional recurrence and distant metastases

**Fig. 2:**
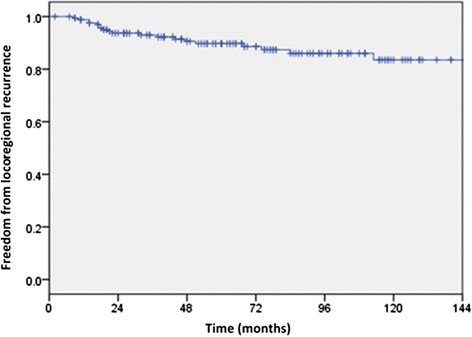
Kaplan-Meier estimates of freedom from locoregional recurrence (FFLRR). Estimated actuarial rates of FFLRR at 5 and 10 years were 90 (95 % CI 85, 95) and 83.5 % (95 % CI 75.5, 91.5)

LRR as a component of first recurrence occurred in 20 patients and is illustrated in Fig. 2. The estimated actuarial rates of FFLRR at 5 and 10 years were 90 (95 % CI 85, 95) and 83.5 % (95 % CI 75.5, 91.5). The median time to LRR in those who experienced it was 27 months (range 9, 147).

DM as a component of first recurrence occurred in 48 patients. The estimated 5 and 10 year actuarial rates of FFDM were 74.5 (95 % CI 67.5, 81.5) and 64 % (95 % CI 53, 75) respectively.

### Disease free and overall survival

**Fig. 3:**
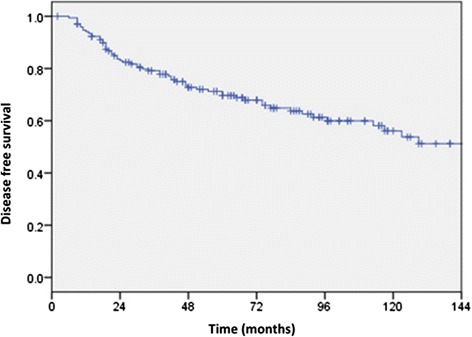
Kaplan-Meier estimates of disease free survival (DFS). Estimated 5 and 10 year DFS were 69.5 (95 % CI 62.5, 76.5) and 56 % (95 % CI 48, 64) respectively

**Fig. 4:**
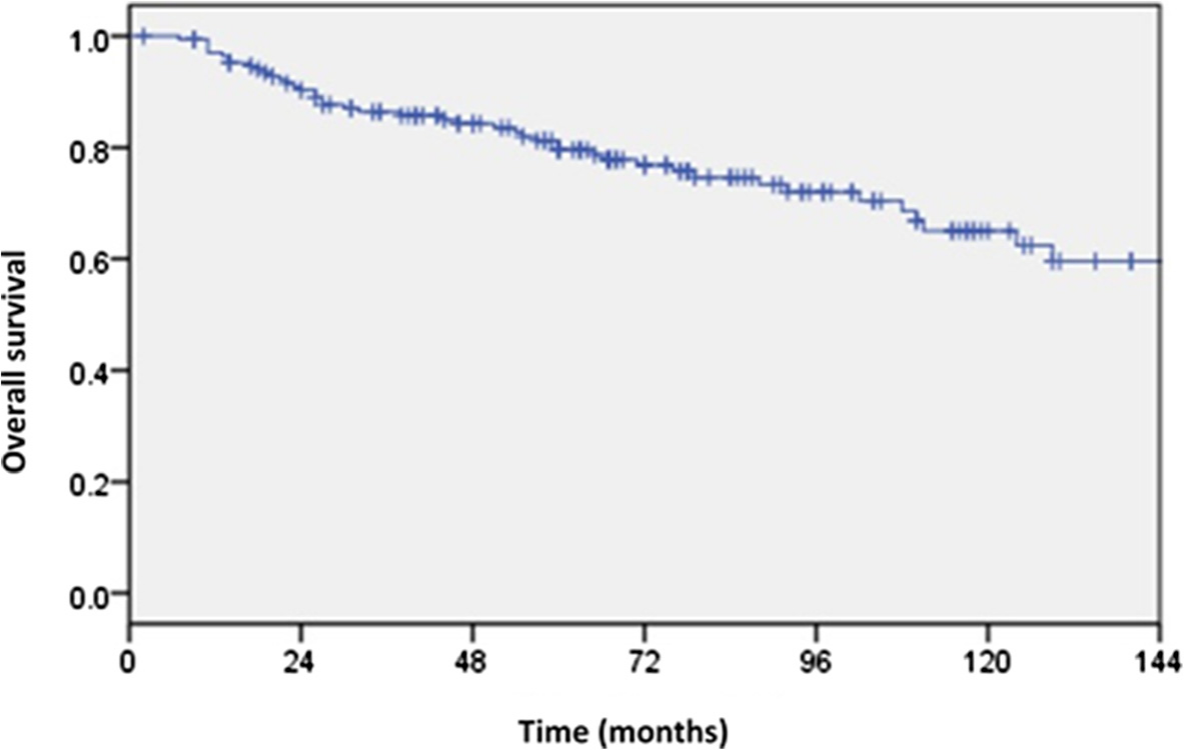
Kaplan-Meier estimates of overall survival (OS). The estimated OS at 5 and 10 years were 79.5 (95 % CI 73, 86) and 65 % (95 % CI 55, 75) respectively

Forty-seven patients died during the follow up period and the majority of deaths (*n* = 40) were related to breast cancer with 2 from other causes and 5 from causes unknown. A further 13 patients experienced recurrence but were alive at the time of last follow up. DFS is represented in Fig. 3 and OS in Fig. 4. The estimated 5 and 10 year DFS were 69.5 (95 % CI 62.5, 76.5) and 56 % (95 % CI 48, 64) respectively. The estimated OS at 5 and 10 years were 79.5 (95 % CI 73, 86) and 65 % (95 % CI 55, 75) respectively. Median time to death in those who experienced it was 44 months (range 7, 177).

### Predictive factors for freedom from locoregional recurrence and overall survival

Uni- and multivariate analysis are summarised in Table 2. On univariate analysis clinical stage II (versus clinical stage III) and estrogen receptor positivity predicted for FFLRR. On multivariate analysis they both remained statistically significant. On univariate analysis age less than 50 years, clinical stage II, cN0, ypN0 and trastuzumab use were all statistically significant prognostic factors. On multivariable analysis estrogen receptor positivity and ypN0 stage remained statistically significant.

**Table 2:** Univariate and multivariate analysis for freedom from locoregional recurrence (FFLRR) and overall survival (OS)

			Freedom from locoregional recurrence					Overall Survival				
			Univariate		Multivariate			Univariate		Multivariate		
Factor		Number	5 yr FFLRR (%)	*p*-value	HR	(95 % CI)	*p*-value	5 yr OS (%)	*p*-value	HR	(95 % CI)	*p*-value
Age	<50	92	93.9	0.35				83.7	0.02	1.90	(0.97-3.7)	0.06
	> = 50	80	84.9					75				
Combined c stage	II	111	94.5	0.00	5.51	(1.35-22.5)	0.02	88.8	0.01	1.25	(0.58-2.7)	0.56
	III	61	81					62.7				
cN stage	o	69	93.6	0.21	1.00	(0.27-3.71)	1.00	87.7	0.00	2.05	(0.92-4.55)	0.08
	1	101	87					74.2				
Inflammatory disease	No	153	90.2	0.09				81.9	0.10	2.50	(0.96-6.52)	0.06
	Yes	19	84.7					58.5				
Grade	1 or 2	99	89.8	0.82				80.8	0.39			
	3	73	91.1					78.3				
Estrogen receptor	Negative	64	80	0.05	5.52	(1.84-16.55)	0.00	72.2	0.14	2.50	(1.22-5.12)	0.01
	Positive	101	95.1					84.9				
Chemotherapy regimen	Anthracyc	97	90.7	0.22				76.9	0.26			
	Anthracyc	54	85.9					86.5				
Surgery	BCS	90	95.8	0.04	3.26	(0.97-10.94)	0.06	81.9	0.76			
	MRM	82	82.8					75.8				
Combined yp stage	0 - II	163	89.9	0.97				81	0.09			
	III	9	87.5					55.6				
pCR	No	143	88.4	0.18				77.2	0.13			
	Yes	28	95.7					91.7				
ypN stage	0	95	89.3	0.94	1.30	(0.42-3.95)	0.65	88.9	0.00	3.17	(1.33-7.15)	0.01
	1	77	90.1					69				
>1 node involved	No	138	90.5	0.33				82.3	0.06	1.26	(0.58-2.75)	0.56
	Yes	34	86.5					69.3				
Trastuzumab use	No	146	91.7	0.09			0.06	77.1	0.03	0.21	(0.03-1.53)	0.12
	Yes	26	79.3					93.3				

## Discussion

The role of post-operative radiotherapy following neoadjuvant chemotherapy for early breast cancer is uncertain and practice is far from uniform [7, 10]. A rationale for a local radiotherapy only approach can be made from recurrence data from landmark non-NAC post mastectomy randomised control trials reporting the vast majority of LRR as local, and from both prospective and retrospective studies reporting low locoregional recurrence rates in selected groups post NAC and mastectomy without adjuvant radiotherapy [5, 11–13]. The recognised morbidity of post-operative dedicated regional radiotherapy in addition to local radiotherapy may hence outweigh its benefit in some. Patient data from our cohort suggests that selection for this radiotherapy program was dominated by patients who had a good response to NAC with almost all converting to combined yp stage 0, I or II and only 20 % having more than 1 lymph node involved.

Cross study comparison is difficult. The majority of modern series exploring the exclusion of post-operative radiotherapy after NAC are conducted exclusively in ypN0 or pCR groups with arguably better outcomes [3, 5, 6, 14]. Recurrence data from early NAC trials, prior to routine regional radiotherapy, report similar recurrence patterns in those who received breast conservation therapy and local radiotherapy despite consisting of only combined clinical stage I and II disease [11].

The subdata analysis unsurprisingly suggested that patients of earlier clinical stage, negative nodes and estrogen receptor positive phenotype faired best. The overall number of patients with a pathologically complete response and pathological stage III disease were likely too small for meaningful predictive analysis. The suggestion that breast conservation was favourable relative to mastectomy for FFLRR almost certainly reflects more advanced clinical disease receiving more intense therapy inclusive of mastectomy.

Isolated local recurrence (*n* = 2), isolated regional recurrence (*n* = 3) and locoregional without distant recurrence (*n* = 3) were uncommon. However, as a component of first failure regional recurrence was perhaps more frequent than expected (*n* = 15) with the supraclavicular fossa involved in nine of the fifteen regional recurrences (including all three isolated regional recurrences). This site would typically be covered by routine regional radiotherapy. It is not possible to discern the temporal relationship of combined site failures from this study but it is certainly tempting to hypothesize that distant recurrence in some patients may represent sequential seeding from an unsterilized regional site. In which case, the addition of regional radiotherapy may have impacted on disease outcomes. The National Surgical Adjuvant Breast and Bowel Project (NSABP) B-51 randomised controlled trial will assist in addressing this question for patients who convert to ypN0 status post NAC.

The premise of omitting regional radiotherapy in this group of patients was that a potentially modest improvement in disease specific outcomes may be outweighed by treatment related toxicity. Whilst there is a theoretical increased risk of brachial plexopathy, thyroid dysfunction and potential second malignancy with regional radiotherapy in addition to chest or conserved breast the absolute increased risk is likely to be low and there is minimal literature to aid. Upper limb lymphedema is a commonly cited concern of regional radiotherapy but data from two recently published, high quality randomised control trials using 3D conformal techniques reported lower than anticipated rates [15, 16]. Comparing local radiotherapy with and without regional radiotherapy in the non-NAC, pN1, post-operative setting, the European Organisation for Research and Treatment of Cancer trial 22922/10925 and the National Cancer Institute of Cancer MA-20 trial reported 12 and 8.4 % lymphedema rates with regional radiotherapy, versus 10.5 and 4.5 % without regional radiotherapy at median follow up of 10.9 and 9.5 years respectively [15, 16].

This study is a retrospective analysis of prospectively collected data and as such has weaknesses. There was much heterogeneity of chemotherapy, HER2 amplification testing and targeted therapy over the data collection period and these areas have evolved considerably. Poorly represented subgroups in this cohort are cN stage >1, ypN stage > 1, combined yp stage > II, inflammatory breast cancer and those with non-ductal histology.

## Conclusion

Local radiotherapy following NAC and oncological resection for clinical stage II and III breast cancer may be a reasonable option in selected patients considered at low risk of harbouring regional disease. Such a hypothesis however requires confirmation from high quality, randomised control trials and recruitment into studies, such as NSABP B-51 for those converting to ypN0, is encouraged. Whilst distant recurrence is the dominant relapse pattern, regional recurrence as a component of first failure was not uncommon.

## Abbreviations

AJCC, American Joint Committee of Cancer; CT, computed tomography; DFS, disease free survival; DM, distant metastasis; ER, estrogen receptor; FFDM, freedom from distant metastasis; FFLRR, freedom from locoregional recurrence; HER2, human epidermal receptor 2; LR, local recurrence; LRR, locoregional recurrence; NAC, neoadjuvant chemotherapy; OS, overall survival; pCR, pathological complete response; RR, regional recurrence; SCF, supraclavicular fossa
